# Enrichment of Elevated Plasma F_2t_-Isoprostane Levels in Individuals with Autism Who Are Stratified by Presence of Gastrointestinal Dysfunction

**DOI:** 10.1371/journal.pone.0068444

**Published:** 2013-07-03

**Authors:** Phillip Gorrindo, Christianne Joy Lane, Evon Batey Lee, BethAnn McLaughlin, Pat Levitt

**Affiliations:** 1 Medical Scientist Training Program, Vanderbilt University, Nashville, Tennessee, United States of America; 2 Department of Biostatistics, Keck School of Medicine, University of Southern California, Los Angeles, California, United States of America; 3 Kennedy Center for Research on Human Development, Vanderbilt University, Nashville, Tennessee, United States of America; 4 Department of Pediatrics, Vanderbilt University, Nashville, Tennessee, United States of America; 5 Department of Psychology, Vanderbilt University, Nashville, Tennessee, United States of America; 6 Department of Psychiatry, Vanderbilt University, Nashville, Tennessee, United States of America; 7 Department of Neurology, Vanderbilt University, Nashville, Tennessee, United States of America; 8 Department of Pharmacology, Vanderbilt University, Nashville, Tennessee, United States of America; 9 J.B. Marshall Neurovascular Laboratory, Vanderbilt University, Nashville, Tennessee, United States of America; 10 Department of Pediatrics, Keck School of Medicine, University of Southern California, Los Angeles, California, United States of America; 11 Institute for the Developing Mind, Children's Hospital Los Angeles, Los Angeles, California, United States of America; Institute of Genetics and Biophysics, Italy

## Abstract

Etiology is unknown in the majority of individuals with autism spectrum disorder (ASD). One strategy to investigate pathogenesis is to stratify this heterogeneous disorder based on a prominent phenotypic feature that enriches for homogeneity within population strata. Co-occurring gastrointestinal dysfunction (GID) characterizes a subset of children with ASD. Our current objective was to investigate a potential pathophysiological measure to test the hypothesis that children with both ASD and GID have a more severe metabolic dysfunction than children with ASD-only, given that the highly metabolically active brain and gastrointestinal system may additively contribute measurable impairment. Plasma levels of F_2t_-Isoprostanes (F_2_-IsoPs), a gold standard biomarker of oxidative stress, were measured in 87 children in four groups: ASD-GID, ASD-only, GID-only and Unaffected. F_2_-IsoP levels were elevated in all 3 clinical groups compared to the Unaffected group, with the ASD-GID group significantly elevated above the ASD-only group (mean, SD in pg/mg: ASD-GID 53.6, 24.4; ASD-only 36.5, 13.3; *p* = 0.007). Adjusting for age, sex, and triglyceride levels, F_2_-IsoP levels remained significantly different between study groups, with a moderate effect size of η_p_^2^ = 0.187 (*p* = 0.001). Elevation in peripheral oxidative stress is consistent with, and may contribute to, the more severe functional impairments in the ASD-GID group. With unique medical, metabolic, and behavioral features in children with ASD-GID, the present findings serve as a compelling rationale for both individualized approaches to clinical care and integrated studies of biomarker enrichment in ASD subgroups that may better address the complex etiology of ASD.

## Introduction

Although the etiology and pathobiology of autism spectrum disorder (ASD) remain elusive, an abundance of genetic studies [1], [2] strongly supports rare *de novo* mutations and common heritability of risk for developing ASD [3], [4]. A persistent challenge for the field has been characterizing and understanding that even in the face of identical genetic etiologies, pronounced clinical heterogeneity of individuals on the spectrum exists. This is likely due to the complexity of pathogenic mechanisms and distinct behavioral and medical manifestations of the disorder. Disorder heterogeneity challenges the field in efforts both to understand altered brain neurobiology [5], and to develop clinical best practices [6], [7]. One strategy to address heterogeneity is to stratify populations of affected individuals based on shared phenotypic features. Effective stratification thereby yields subpopulations with greater intra-group homogeneity by enriching for subtype-specific phenotypes that may reflect shared underlying pathophysiology and clinical presentation.

This approach to phenotypic stratification has previously proven effective and analytically important. Eye-tracking measures demonstrated a relationship between social disability and preferential visual scanning of non-social aspects of visual scenes; however this relationship was reversed in subgroups of individuals with ASD stratified by cognitive function [8]. Moreover, association between a genetic variant and ASD met Bonferroni-corrected genome-wide significance only when patient gender was considered [9]. Neuroimaging studies using genetic enrichment of common ASD risk alleles have been used to identify unique functional and structural networks within phenotypic strata, thus reducing overall subject heterogeneity [5], [10]. We have reported genetic signal enrichment in individuals with ASD and co-occurring gastrointestinal dysfunction (GID) compared to individuals with ASD but who are not diagnosed with co-occurring GID [6]. This finding supports our hypothesis that pleiotropic expression of an ASD-associated genetic variant in the brain and gastrointestinal system confers a shared risk for parallel dysfunction in both organ systems. However, although the utility of phenotypic stratification is clear, reports leveraging phenotypic variability in study design and analysis remain limited.

GID is common in children with ASD [11], but the field is just beginning to understand its contribution to ASD heterogeneity and severity of core symptoms. As with efforts to study other medical co-morbidities, there have been challenges in understanding the intersection of GID and ASD: GID can be mediated and influenced by behavior, medications, and diet, complicating determination of pathophysiological origin; an unsubstantiated view that parents over-report GID in their children with ASD; a lack of evidence for the efficacy of specialized diets; a lack of prospective reports on GID that include evaluations by pediatric gastroenterologists; and a lack of consensus among retrospective studies regarding GID prevalence (reported prevalence rates ranging from 9 to 70%) [12]–[16]. We reported that 41% of children in the Autism Genetics Resources Exchange (AGRE) cohort had parent-reported GID [6], a finding that was recently replicated [17]. Subsequently, in the first prospective study of children with co-occurring ASD and GID (ASD-GID) [18], we reported four noteworthy findings: 30% of ASD-GID children had minimal to no verbal abilities, compared to 6.7% in the ASD-only group; an elevated mean T-score of 89 for ASD-GID, compared to 77 for ASD-only, on the Social Responsiveness Scale (SRS); no differences in the nature of diets or limited food group diversity between children with ASD, with or without GID; children in both the ASD-GID and the ASD-only groups were prescribed medications with potential gastrointestinal side effects, but no difference in prevalence of these medications between groups was detected. These data support a pronounced clinical phenotype of children with co-occurring ASD and GID, a percept that has been noted by expert clinicians [11], [19], suggesting these children with comorbid GID may be more severely impacted by their disease.

Based on several features of ASD and GID, we put forth a convergent hypothesis suggesting the possibility that pronounced metabolic dysfunction characterizes a subgroup of children with ASD-GID. First, both the brain and gastrointestinal system are highly metabolically active, with a likely shared susceptibility to insults that produce parallel disturbances in function. Second, approximately 7% of individuals with ASD are reported to have mitochondrial dysfunction [20]. Third, physiological measures and genetic data support the conclusion that a subgroup of children with ASD exhibit increased levels of peripheral oxidative stress [21]–[25]. The ASD-GID, ASD-only and GID-only subgroups thus provide a comparative opportunity to examine stratification based on oxidative stress status.

Valid measurements of oxidative stress in clinical populations are challenged by multiple factors, each of which the current study attempted to address. First, of the diverse strategies for quantifying oxidative stress and metabolic dysfunction, we elected to measure plasma F_2t_-Isoprostanes (F_2_-IsoPs). Isoprostanes are prostaglandin-like molecules formed *in vivo* via the non-enzymatic free radical-mediated oxidation of arachidonic acid. F_2_-IsoPs are biologically inert and long lived, and the NIEHS commissioned Biomarkers of Oxidative Stress Studies demonstrated these species to be the most sensitive indicators of redox dysfunction, establishing them as the gold standard measure of oxidative stress [26]–[28]. Levels of F_2_-IsoPs are elevated in a variety of disorders across multiple organ systems [29]–[31]. Second, we collected data on the largest sample size to date in order to yield sufficient statistical power to address potential heterogeneity and variance across comparative groups. Third, we included three clinical populations (ASD-GID, ASD-only, GID-only) and an Unaffected control group, which enabled us to perform functionally meaningful stratification. We report here that although all three clinical groups exhibited elevated oxidative stress levels, there is a previously undocumented extreme subgroup of children in the ASD-GID group who may be at particularly high risk for metabolic dysfunction.

## Materials and Methods

### Participants, Study Procedures and Enrollment Criteria

Children aged 5 to 18 years were recruited at Vanderbilt University in Nashville, Tennessee into three groups: co-occurring ASD and GID, ASD without any GID, and GID without any ASD. Data presented here are from a subset of individuals who participated in a larger, previously reported study of co-occurring ASD and GID that includes a rich clinical characterization of the three groups studied here [18]. Table 1 includes summary clinical data for the subset of children analyzed in the present study.

**Table 1:** Basic Characteristics of Study Participants.

	ASD-GID	ASD-only	GID-only	Unaffected
	(n = 27)	(n = 29)	(n = 21)	(n = 10)
Age in years, mean (SD)	11.4 (3.2)	13.1 (3.3)	11.3 (3.3)	11.3 (2.0)
Male sex,% (n)	85.2 (23)	89.7 (26)	47.6 (10)	100 (10)
Ethnicity and race,% (n)				
Hispanic	3.7 (1)	0 (0)	4.8 (1)	0 (0)
Non-Hispanic white	85.2 (23)	89.7 (26)	85.7 (18)	100 (10)
Non-Hispanic black	7.4 (2)	6.9 (2)	9.5 (2)	0 (0)
Non-Hispanic other	3.7 (1)	3.4 (1)	0 (0)	0 (0)
Gastrointestinal Diagnosis,% (n) *		n/a		n/a
Functional Constipation	70.4 (19)		33.3 (7)	
Reflux	18.5 (5)		38.1 (8)	
Irritable Bowel Syndrome	3.7 (1)		19.0 (4)	
Functional Abdominal Pain	3.7 (1)		9.5 (2)	
Other	7.4 (2)		23.8 (5)	

* Note: Some children had more than one diagnosis, thus percentages sum to greater than 100%.

To confirm ASD diagnoses, children in both ASD groups were assessed with the Autism Diagnostic Observation Schedule (ADOS). To characterize GID, children in both GID groups were assessed by either parent report on the Questionnaire on Pediatric Gastrointestinal Symptoms – Rome III (QPGS), a research-validated instrument that categorizes gastrointestinal signs and symptoms into functional gastrointestinal disorders according to Rome-III criteria [32], [33], or by a clinician who specializes in caring for children with GID and who followed the clinical guidelines of the North American Society for Pediatric Gastroenterology, Hepatology and Nutrition (NASPGHAN). We have previously reported the details of these GID characterizations, and shown that in the larger source cohort, the concordance for presence of any type of functional gastrointestinal disorder in children when reported by parents on the QPGS compared to clinician examination exceeds 90% [18]. Parents in the ASD-only group completed the QPGS, to ensure the ASD-only group was devoid of any latent GID that was not identified at study enrollment. Parents in the GID-only group completed the Social Responsiveness Scale, a research-validated instrument of social impairment that is sensitive in a non-ASD population [34], but also correlates strongly with ASD diagnoses [35], to ensure the GID-only group was devoid of any latent social impairment that was not identified at study enrollment.

Exclusion criteria included severe sensory or motor impairment, neurodevelopmental disorders of known etiology (Rett Syndrome, Tuberous Sclerosis, Down Syndrome, Phenylketonuria, 22Q Deletion Syndrome, Fragile X Syndrome and Neurofibromatosis), gestational age less than 36 or greater than 42 weeks, and birth weight less than 2500 grams. Inclusion criteria included age between 5 and 18 years at enrollment, meeting ASD criteria on the ADOS (for the ASD groups), and gastrointestinal signs and symptoms that had lasted more than a month (for the GID groups).

### Control Samples

For an unaffected control comparison group, plasma samples from 10 children were contributed by the laboratory of Dr. Kathryn Edwards (Vanderbilt University). These samples came from participants in an unrelated influenza vaccine study who were recruited from the same geographic region as children in the clinical groups. Inclusion criteria included: parent reported absence of verbal or motor delays greater than 10% of their peers, parent report of the child being in good overall health, and the child's ability to understand and verbally assent to the blood draw procedure. These ten participants were not assessed with ASD- or GID-specific instruments.

### Blood Samples and Plasma Preparation

Whole blood (4 ml) was drawn via peripheral venipuncture from study participants into EDTA-coated blood tubes (BD, Franklin Lakes, NJ) and centrifuged at 1,000 *g* for 10 m at 4°C. Blood collection in the presence of EDTA is the preferred method for minimizing *ex vivo* oxidation and maintaining stability of F_2_-IsoPs, while being compatible with measurement of blood lipids [26]. Supernatant plasma was drawn off the sample and distributed (0.5 to 1 ml aliquots) before storing at −80°C for later analysis. If blood was not centrifuged immediately after venipuncture, it was placed on ice within 15 m of venipuncture. Plasma was frozen within 1 h of blood draw, and kept at −80°C until further processing for measurement of F_2_-IsoPs.

### Measurement of F_2_-Isoprostanes in Plasma

Our method of measuring F_2_-IsoPs has been previously described [26], [36], and is stated here in brief. One ml of plasma was added to 1.0 ng of [^2^H_4_]-15-F_2t_-IsoP (also known as [^2^H_4_]-8-iso-PGF_2a_; Cayman Chemical, Ann Arbor, MI, USA) internal standard. The solution then was processed such that derivatized F_2_-IsoP compounds were isolated, dried, and re-dissolved for Gas Chromatography/Mass Spectrometry (GC/MS) analysis [26], [37]. GC/Negative Ion Chemical Ionization-MS (GC/NICI-MS) was performed with an Agilent 5973 Inert Mass Selective Detector coupled with an Agilent 6890n Network GC system (Agilent Labs, Torrance, CA, USA). Levels of endogenous F_2_-IsoPs in plasma were calculated from the ratio of intensities of ion *m/z* 569 (major ion generated by the F_2_-IsoPs derivatives) to ion *m/z* 573 (corresponding ion generated by the internal standard). The precision of this assay in biologic fluids is ±6%, and the accuracy is 94% [26], [37]. Data are reported as pg of F_2_-IsoPs per mg of total protein in the sample. GC/NICI-MS measurements and analyses were completed at the Vanderbilt University Medical Center Eicosanoid Core [26]. Group identity of each sample was obscured for the duration of sample measurement and analysis.

### Measurement of Triglycerides in Plasma

The levels of F_2_-IsoPs that accumulate in blood through lipid peroxidation can be influenced by variations in blood lipid concentration *a priori* [38]. In order to appropriately co-vary for individual subject differences in blood lipids, plasma samples were run for low density, very low density and high density lipids using a commercially available bioassay from BioVision. In the assay, cholesterol oxidase specifically recognizes free cholesterol and produces products that react with probe to generate color (570 nm) and fluorescence (Ex/Em = 538/587 nm). Cholesterol esterase hydrolyzes cholesteryl ester into free cholesterol, therefore, cholesterol ester and free cholesterol can be detected separately in the presence and absence of cholesterol esterase in the reactions. Inter-run reliability was assessed by using a single subject frozen aliquot of plasma, which demonstrated <5% variability between assays.

### Data Analysis

Study data were managed using REDCap, a secure, research-oriented, web-based application [39]. Statistical analyses were computed using SPSS version 19.0.1 (IBM, Somers, NY) and the plot was generated using Prism version 5.0d (GraphPad, La Jolla, CA). Age was described by mean and standard deviation, and sex, ethnicity and race were described by percent of group. Gastrointestinal dysfunction was treated as a binary variable (present or absent), without distinction for specific type of GID. There is, however, substantial homogeneity of type of GID within the larger source cohort (i.e., 85% constipation) [18]. For F_2_-IsoP and triglyceride levels, mean and standard deviation (SD) descriptive statistics were used. F_2_-IsoP levels were compared with a one-way ANOVA test with Tukey's HSD post-hoc pairwise comparisons. A Pearson's correlation coefficient (r) was calculated to assess correlation between F_2_-IsoP and triglyceride levels. To adjust for possible confounding effects of age, sex, and triglyceride levels on F_2_-IsoP levels, we performed an ANCOVA with these factors as covariates in a model. The resulting partial eta squared (η_p_^2^) and adjusted mean and SD values are reported. Distribution of residuals generated by this model were assessed using a Kolmogorov-Smirnov test of normality. SRS T-scores are normally distributed [34], and were therefore described with mean (SD) and compared with a one-way ANOVA with Tukey's HSD post-hoc test. For all statistical analyses, a two-tailed *p* value of less than 0.05 was considered significant.

### Ethics Statement

The research protocol was approved by the Vanderbilt University Institutional Review Board, and written informed consent was obtained from parents of participants.

## Results

### Participant Characteristics

Eighty-seven children were recruited for this study (Table 1; group sizes: ASD-GID = 27, ASD-only = 29, GID-only = 21, Unaffected = 10). In general, participants were young adolescents (range of mean ages among groups: 11.3 – 13.1 y), male (range among groups: 47.6 − 100%), and of non-Hispanic white self-reported ethnicity and race (range among groups: 85.2 − 100%). All children in both ASD groups included in the current study met classification criteria for autism on the ADOS, using the revised scoring algorithm [40]. No child had a seizure disorder. Children in both GID groups had their gastrointestinal signs and symptoms characterized by either a research-validated parent-reported instrument, the QPGS [41], a history and physical exam by a clinician with gastrointestinal expertise, or both. We previously reported 92% agreement between parent report and clinical evaluation for presence of GI complaints in a larger source cohort [18]. For children in the ASD-GID group, 15% were evaluated only by a clinician, 22% only by the parent reported QPGS, and 63% by both. For children in the GID-only group, 24% were evaluated only by a clinician, and 76% were evaluated by both a clinician and QPGS. Of the 48 children with GID (ASD-GID and GID-only children), 95.8% were evaluated by a pediatric gastroenterologist and 4.2% were evaluated by a nurse practitioner who works in close association with a pediatric gastroenterologist. All children in the ASD-only group had a complete QPGS screen, and did not meet criteria for any GID classification (except two children who met criteria for fecal incontinence, which is confounded by toilet training in younger children, and thus both participants were still included in the ASD-only group). Gastrointestinal diagnoses for the ASD-GID and GID-only groups are listed in Table 1. The clinical data in this report come from children who participated in a study that has been previously published [18]; of the ASD-only, GID-only and ASD-GID groups described in the present biomarker study, 77% were also included in the previous study.

### F_2_-IsoP Levels in Plasma

**Figure 1:**
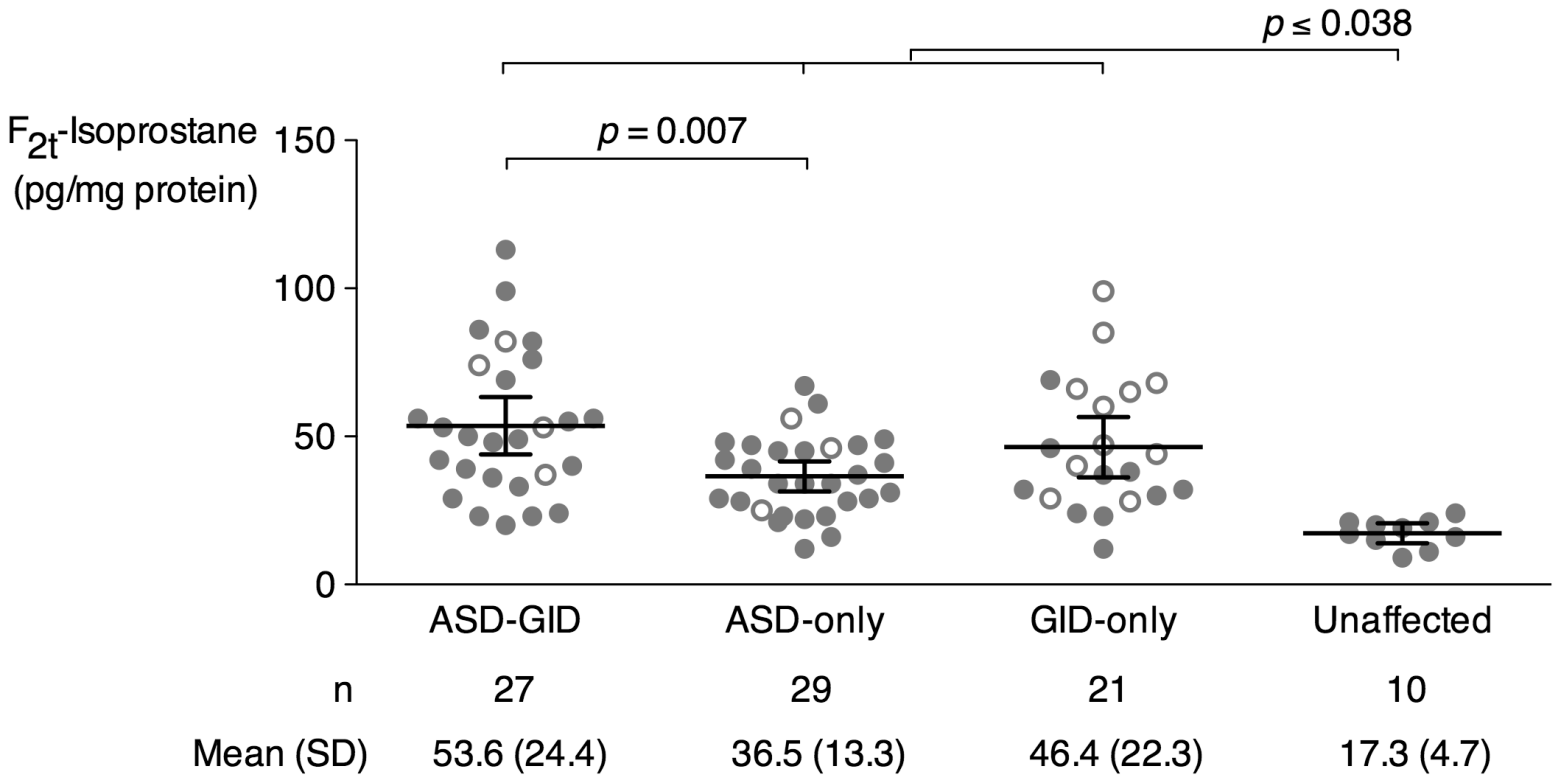
Plasma F_2t_-Isoprostane levels among four study groups. Data are plotted as mean (SD). Open circles are female participants; closed circles are male participants.

F_2_-IsoPs were measured in plasma from all participants (Figure 1). In a one-way analysis of variance (ANOVA), the ASD-GID group F_2_-IsoP level was significantly elevated above that in the ASD-only group (ASD-GID mean 53.6 pg/mg total protein, SD 24.4; ASD-only 36.5 pg/mg, 13.3; *p* = 0.007). The ASD-only and GID-only group levels were not significantly different (GID-only 46.4 pg/mg, 22.3), nor were the ASD-GID and GID-only group levels. The Unaffected group F_2_-IsoP level was significantly lower compared to all other groups (Unaffected 17.3 pg/mg, 4.7; *p*≤0.038 for all Unaffected pairwise comparisons). F_2_-IsoP levels in the Unaffected control group were comparable to levels reported in normal healthy humans [26], [42].

### Triglyceride Levels in Plasma

As a potential biosynthetic source of F_2_-IsoPs, total triglyceride levels also were measured in plasma samples. The group levels for ASD-GID, ASD-only, and GID-only were not significantly different (per group, mean, SD in mg/dl were ASD-GID 133.2, 28.7; ASD-only 129.0, 23.4; GID-only 134.8, 23.7). ASD-GID, ASD-only, and GID-only all had significantly elevated group triglyceride levels compared to Unaffected control children (85.6 mg/dl, 21.2). Triglycerides were not measured for one sample each from the ASD-only and GID-only groups, due to insufficient sample amount.

### F_2_-IsoP Levels Adjusted for Covariates

Because of possible confounding effects of age, sex, and triglyceride levels on the primary outcome measure of F_2_-IsoP levels, we performed an adjusted analysis. Notably, our unadjusted analysis showed significantly different F_2_-IsoP levels (Figure 1), but our unadjusted analysis of triglyceride levels also showed significantly different levels between the Unaffected group compared to the three other clinical groups in a similar pattern to that seen in unadjusted F_2_-IsoP levels. For all samples, triglyceride levels significantly correlated with F_2_-IsoP levels (*p* = 0.001, Pearson's r = 0.36). To clarify the relationship between study group and F_2_-IsoP levels, we performed an analysis of covariance (ANCOVA) with age, sex, and triglyceride level as covariates. Representing a moderate effect size, we found a partial eta squared (η_p_^2^) value of 0.187 for the relationship between study group and adjusted F_2_-IsoP levels (Table 2; *p* = 0.001). This indicates that, when accounting for age, sex, and triglyceride level, the study group identity accounts for 18.7% of the overall variance in F_2_-IsoP levels. Table 3 compares the unadjusted and ANCOVA-adjusted mean F_2_-IsoP levels. Residuals generated by this model were normally distributed (*p* = 0.15).

**Table 2:** Analysis of Covariance — Relative Contribution of Covariates to F_2t_-Isoprostane Levels.

	Partial Eta Squared (η_p_^2^)	*p* value
Age	0.009	NS
Sex	0.087	0.008
Triglycerides *	0.051	0.044
Study Group	0.187	0.001

* n = 85 (Triglyceride data not available for 1 sample each from ASD-only and GID-only groups).

**Table 3:** Analysis of Covariance — Age-, Sex-, and Triglyceride-Adjusted F_2t_-Isoprostane Levels.

	F_2t_-Isoprostane (pg/mg protein), mean (95% CI)	
	Unadjusted	Adjusted
ASD-GID	53.6 (46.2–60.9)	53.0 (46.2–59.9)
ASD-only	36.8 (29.4 – 43.6)	38.4 (31.5 – 45.2)
GID-only	46.4 (38.0–54.7)	37.6 (28.9–46.4)
Unaffected	17.3 (5.2–29.4)	26.6 (13.4–39.8)

### Social Impairment

To gain additional insight into the complex clinical phenotype of children with co-occurring ASD and GID, we quantitatively assessed social impairment using the SRS, a research-validated and parent-reported index of reciprocal social behavior in children. SRS T-Scores are normalized to a mean of 50 in typically-developing children, and higher T-Scores indicate more severe social impairment. Consistent with our previous report [18], the group mean T-Scores for ASD-GID, ASD-only, and GID-only were all significantly different (per group, mean, standard deviation of T-scores were: ASD-GID 90.6, 13.4; ASD-only 75.7, 17.1; GID-only 51.4, 14.1; *p*≤0.002 for all three pair-wise comparisons), with the most severe impairment in the ASD-GID group. SRS data were not available for four children each in the ASD-GID and GID-only groups.

## Discussion

In this study of children with ASD who were stratified by presence of a co-occurring medical condition – gastrointestinal dysfunction – there was a significant elevation of plasma F_2_-IsoP levels in the ASD-GID compared to the ASD-only group. Adjusting for age, sex, and triglyceride level showed a significant difference in F_2_-IsoP levels between groups, with 18.7% of the variance in F_2_-IsoP levels attributable to study group (η_p_^2^ = 0.187). Moreover, in parallel to the medical and physiological disruptions seen in the ASD-GID group, these children also demonstrated significantly more impaired social function based on SRS scores, compared to ASD-only and GID-only children. These findings are consistent with our hypothesis that individuals with co-occurring ASD and GID may exhibit clinical phenotypes that are sufficiently distinct from children with ASD but without GID to warrant more individualized approaches to medical and ASD-specific interventions. Moreover, the subpopulation of children in the ASD-GID group with extremely elevated F_2_-IsoP levels above those in both the GID-only and ASD-only groups is consistent with a hypothesis that dysfunctional biology in parallel organ systems – in this case, oxidative stress in the brain and gastrointestinal system, both of which are highly metabolically active – may have additive effects in disrupting behavioral and biological functions of affected individuals. Our findings suggest that analysis of peripheral biomarkers in future studies of individuals with ASD, or even young siblings who are at greater risk, might also benefit from considering, and possibly stratifying upon, presence of medical and behavioral comorbidities. In ASD, this can include sleep disturbances, seizures, sensory and motor impairments and abnormal emotional regulation [7].

The F_2_-IsoPs elevation in ASD-only compared to Unaffected controls is consistent with two previous smaller studies, which showed elevation of urinary F_2_-IsoPs relative to controls [43], [44]. Neither study, however, examined F_2_-IsoP levels in the context of social impairment severity, co-occurring medical condition or triglyceride levels. Ming and colleagues did report on parent-reported GID symptoms in their cohort, suggesting an interesting possible re-analysis in which their cohort could be stratified by presence of GID. The study by Yao and colleagues does not mention assessing for GID in their population, but, interestingly, their data show a subpopulation of children in the ASD group with much higher F_2_-IsoP levels, which might be accounted for by the presence of GID or other medical condition.

A potential difficulty in interpreting the data presented here is that we have used one method, among multiple possible methods employed in different laboratories, to assess oxidative stress status in our study participants. There are several important aspects of our preferred method that are worth noting. Our method of measuring F_2_-IsoP levels relies on rapid processing of samples, no sample refreezing, and EDTA present during blood collection. These parameters together promote a stable sample for GC/NICI-MS analysis. Butylated hydroxytoluene (BHT) or other antioxidants may be required for sandwich ELISA detection methods, in which samples are not processed rapidly or collected in EDTA. However, BHT is not required when using our collection and measurement protocols. Additionally, all of our assays included a standard plasma sample from one individual to demonstrate consistency of the assay across analytical runs. The detection method used here has been described in detail [26], and was referenced in the NIEHS Biomarkers of Oxidative Stress Study as the gold standard [26]–[28].

Cognitive and adaptive levels of functioning were not assessed in this study, and GID prevalence may by higher among lower functioning individuals with ASD. Future studies would benefit from assessing IQ and adaptive behavior levels among participants, both cross-sectionally, and over the temporal course of GID manifestation, treatment, and developmental trajectory.

With regard to potential caveats of GID diagnoses in the subjects of this study, we note that most participating children were not examined endoscopically, which under certain clinical circumstances provides better characterization of the nature and extent of their GID. However, endoscopy is not standard of care for children for whom functional constipation is the primary diagnosis [19], and endoscopy without clinical indication would subject a child to more than minimal risk, for which our IRB protocol was not approved. In a previous report [18], we noted that when clinically warranted, based on NASPGHAN criteria and clinical judgment, children were evaluated by endoscopy and laboratory tests (complete blood count, comprehensive metabolic panel, erythrocyte sedimentation rate, and celiac screening panel). We also reported that laboratory tests and endoscopy results were clinically benign and unremarkable for all except 7 children (four cases of eosinophilic esophagitis, and one case each of *H. pylori*, celiac disease, and Crohn's disease). In the current report, of these 7 children, one with *H. pylori* and one with eosinophilic esophagitis are included.

Finally, with regard to potential study caveats, we wondered about the possible influence of sex of participants on the data presented here. Statistical analysis by co-variation did not detect group differences in F_2_-IsoP levels based on male-female differences. Although there are more females than males in the GID-only group, group differences in F_2_-IsoP levels were not due to any statistically significant differences in F_2_-IsoP levels between male and female children in our study. Combined, these points demonstrate that the differences in F_2_-IsoP levels are not driven by sex of participants in different study groups. It is also worth noting that because ASD is much more prevalent in males, it was not feasible for our study to include a sex-matched group.

The data presented here contribute to a compelling argument for integrative analyses of clinical manifestations of ASD, co-occurring medical conditions, and biomarkers that are relevant to pathophysiology. Inclusion of children without ASD, but who exhibit comparative medical conditions also will be important. With regard to children with ASD, it will be important for future studies to include, in study design and analysis, factors that may impact physiological measures of metabolic status, such as medications with possible metabolic side effects, diet, seizure history, and sleep status – all of which may contribute to a more complicated medical picture for some children with co-occurring ASD and GID.

Our data provide a novel, integrated biomedical perspective regarding the stratification of children with ASD, and demonstrate the unique features of children with co-occurring ASD and GID. There may be a point of convergence between the present findings regarding the ASD-GID group of children and the growing interest in interactions between the gastrointestinal system and brain, and the influence of peripheral status on CNS function [45], [46]. The accumulating data emphasize the clinical imperative of addressing the pathogenic mechanisms and unique needs of this group of children, concepts that have been emphasized by consensus reports [11], [19]. Data presented here show that ASD-GID children have (1) medically significant gastrointestinal dysfunction, (2) high risk for oxidative stress based on pathophysiological measures of F_2_-IsoP levels, and (3) more severely disrupted social function. From a research perspective, stratification will be essential to understand the context of specific measures. From a clinical perspective, this group of children faces a distinct constellation of challenges and warrants individualized care and interventions. Looking to the future, we suggest that phenotypic stratification in ASD is a generalizable strategy, and will play a key role in attempts to overcome the complexity that has historically challenged efforts to understand and provide the best research-informed care for individuals with ASD.
